# Synergistic effects of micropatterned substrates and transforming growth factor-β1 on differentiation of human mesenchymal stem cells into vascular smooth muscle cells through modulation of Krϋppel-like factor 4

**DOI:** 10.1007/s11626-025-01033-2

**Published:** 2025-05-23

**Authors:** Sakhavat Abolhasani, Davood Fattahi, Yasin Ahmadi, Behnaz Valipour, Majid Ghasemian, Masoumeh Rajabibazl, Khalil Maleki Chollou

**Affiliations:** 1 Department of Basic Sciences and Health, Sarab Faculty of Medical Sciences, Sarab, East Azerbaijan Iran; 2 School of Pharmacy and Biomolecular Sciences, Liverpool John Moores University, James Parsons Building, Byrom Street, Liverpool, L3 3AF UK; 3 Department of Clinical Biochemistry, School of Medicine, Shahid Beheshti University of Medical Sciences, Chamran Highway, Tehran, Iran; 4 Department of Medical Laboratory Science, Faculty of Science, Komar University of Science and Technology, Sulaymaniyah, Kurdistan Region Iraq

**Keywords:** Human mesenchymal stem cells, Vascular smooth muscle cells, Micropatterned substrates, Krϋppel-like factor 4, Myosin heavy chain (MYH11)

## Abstract

The functionality and structural integrity of the cardiovascular system are critically dependent on vascular smooth muscle cells (VSMCs). Human mesenchymal stem cells (hMSCs) have significant potential for differentiating into VSMCs, making them a valuable resource in regenerative medicine and the development of vascular grafts. This study explored the synergistic effects of micropatterned substrates and TGF-β1 on the differentiation of hMSCs into VSMCs. HMSCs were cultured on both micropatterned and flat substrates for a duration of 6 days, with some groups receiving TGF-β1 treatment, after which cell morphology and the expression of specific smooth muscle markers were evaluated through Western blotting, immunofluorescence staining, and RT-qPCR. Results indicated that hMSCs on micropatterned substrates treated with TGF-β1 exhibited significantly elevated protein levels of smooth muscle myosin heavy chain (MYH11) compared with hMSCs on flat substrates without TGF-β1 (*p* < 0.001). Additionally, MYH11 expression was markedly enhanced in samples cultured on micropatterned substrates with TGF-β1. Furthermore, hMSCs treated with TGF-β1 on flat substrates exhibited increased cadherin-11 mRNA expression compared with both micropatterned and flat substrates lacking TGF-β1 (*p* < 0.05). Interestingly, KLF4 protein levels were significantly higher in hMSCs on flat substrates without TGF-β1 compared to those cultured on micropatterned substrates with TGF-β1 treatment (*p* < 0.001). In conclusion, this study demonstrated that the combination of micropatterned substrates and TGF-β1 treatment preferentially enhances MYH11 expression, indicative of advanced smooth muscle cell organization, along with modulating KLF4 levels and upregulating cadherin-11 expression in hMSCs. These findings provide critical insights into the differentiation pathways of MSCs into VSMCs and may inform the design of improved vascular grafts that better replicate the properties of native blood vessels.

**Supplementary Information:**

The online version contains supplementary material available at 10.1007/s11626-025-01033-2.

## Introduction

Vascular smooth muscle cells (VSMCs) are critical components of the vascular system and are predominantly located within the tunica media, which is the intermediate layer of blood vessel walls. These particular cells are integral to the regulation of vascular tone, which in turn affects blood pressure and ensures sufficient tissue perfusion throughout the organism (Liu & Lin 2022). VSMCs can be classified into two primary phenotypes: contractile and synthetic. The contractile phenotype is distinguished by high levels of differentiation, limited proliferation, and robust contractile capabilities, whereas the synthetic phenotype is characterized by enhanced proliferation and increased synthesis of extracellular matrix (ECM) proteins, particularly in response to vascular injury or pathological conditions (Alexander & Owens 2012; Ribeiro et al. 2024).

Recent advances in regenerative medicine have shown that bone marrow-derived mesenchymal stem cells (MSCs) contribute to the repair of injured arteries (Amran et al. 2019; Gu et al. 2018). MSCs are characterized as multipotent progenitor cells capable of differentiating into a wide range of cell types, particularly vascular smooth muscle cells (VSMCs). This ability positions them as promising candidates for innovative therapies aimed at enhancing vascular regeneration and developing tissue-engineered vascular grafts (Amran et al. 2019; Engler et al. 2006). Additionally, the efficacy of MSCs in therapeutic contexts is enhanced by their immunomodulatory properties and their secretion of bioactive factors that facilitate tissue repair and regeneration (Trigo et al. 2024). Furthermore, the stem cell niche, which includes cell-to-cell communication, growth factors, and the ECM, has an impact on the fate of adult stem cells. This niche is characterized by a complex interplay of biochemical signals, intercellular communications, and ECM components that collectively influence stem cell behaviors (Rider et al. 2008; Shirkoohi et al. 2024). From the perspective of tissue engineering, integrating MSCs with biomaterials is critical for creating functional vascular constructs. Scaffolds designed to mimic the natural ECM can significantly enhance cell attachment, proliferation, and differentiation (Ghasemi-Mobarakeh et al. 2015). Recent studies have highlighted the importance of cellular morphology in guiding stem cell fate decisions. Techniques such as micropatterning enable precise controlling cell shape and alignment, which can significantly influence differentiation pathways. Parameters such as substrate stiffness and cytoskeletal tension are known to influence MSC differentiation toward a smooth muscle lineage (H. Li et al. 2012a, b; Parandakh et al. 2019; Scodellaro et al. 2024).

Our previous investigation has demonstrated that manipulating MSC morphology to resemble that of contractile SMCs can enhance their differentiation into VSMCs. Specifically, we found that micropatterned substrates combined with transforming growth factor-β1 (TGF-β1) are potent regulators of this differentiation process (Abolhasani et al. 2020). TGF-β1 is a well-established factor that promotes myogenic differentiation by inducing changes in cell morphology and activating transcriptional programs associated with contractility (13). This process involves the regulation of genes linked to smooth muscle contraction through elements such as the CArG box, which is activated by serum response factor (SRF) along with its coactivator myocardin (MYOCD) and myocardin-related transcription factors (MRTFs) (Hendrix et al. 2005). Conversely, Krϋppel-like factor 4 (KLF4) has been identified as a negative regulator of smooth muscle marker gene expression (Liu et al. 2005). KLF4 inhibits CArG box activation and downregulates SRF and myocardin levels, thereby constraining smooth muscle differentiation (Salmon et al. 2012; Weiser-Evans et al. 2024). Additionally, studies indicate that reducing KLF4 expression during stem cell differentiation can enhance the expression of smooth muscle-specific genes (Aji et al. 2017).

Cell adhesion molecules (CAMs), with a particular emphasis on cadherin, are fundamentally important in the modulation of various cellular activities, including proliferation, differentiation, and migration. Abnormalities in these processes contribute to pathologic smooth muscle cell-driven repair (L. Li et al. 2012a, b; Niessen et al. 2011). Cadherin-11 has been demonstrated to be critical for the expression of smooth muscle proteins and the contractile function within organ systems that comprise smooth muscle cells (SMCs) (Alimperti et al. 2014). The current study hypothesizes that MSCs towards an elongated phenotype will affect the expression levels of CAMs, specifically cadherin-11, consequently promoting the differentiation of VSMCs. In this study, the differentiation of human MSCs (hMSCs) into VSMCs was investigated using smooth muscle myosin heavy chain (MYH11) as a mature marker of smooth muscle-specific contractile proteins. Also, this study investigated the role of KLF4 in the combined effects of micropatterned topography and TGF-β1 on the differentiation of MSCs in VSMCs. We hypothesized that altering the shape of MSCs to mimic the contractile phenotype of VSMCs using microgrooved topography along with TGF-β1 stimulation would improve VSMC differentiation by regulating KLF4 levels and increasing the expression of the mature VSMC marker MYH11. In addition, we investigated whether substrate topography and TGF-β1 play a role in the upregulation of cadherin-11, a cell adhesion molecule essential for VSMC differentiation, in hMSCs with altered phenotypes.

## Materials and methods

### Cell source and culture

The human bone marrow-derived MSCs obtained from the Royan Stem Cell Bank in Tehran, Iran, at passage 3 were well characterized for the surface markers CD105^+^, CD90^+^, CD73^+^, and CD45^–^. Surface antigen information was confirmed via flow cytometry analysis, as stated in the Institute’s data sheet (Supplementary data). The hMSCs were cultured in T-75 culture flasks using a proliferation medium (Fisher Scientific) for expansion without inducing differentiation. The cells were cultured in a humidified incubator at 37 °C with 5% CO_2_, with regular medium changes every 2–3 d. Cell detachment was achieved by washing with phosphate-buffered saline (PBS) and incubation with 0.25% Trypsin–EDTA. The trypsin was neutralized; the cells were centrifuged to form a pellet, and then re-suspended in a fresh medium comprising 89% low-glucose DMEM, 10% FBS, and 1% penicillin–streptomycin. The prepared medium served as the normal medium for further experiments.

### Fabrication of PDMS micropatterned substrates

Patterned polydimethylsiloxane (PDMS) substrates with parallel microgrooves (10 μm wide, 5 μm deep, and 10 μm apart) were fabricated using silicon wafers based on established research that highlights their efficacy in simulating natural ECM. This configuration facilitates the alignment of hMSCs and supports the cytoskeletal reorganization required for SMC differentiation (Yeh et al. 2019). The processes involved in silicon wafer microfabrication and silicone elastomer preparation have been detailed in previous publications (Abolhasani et al. 2020). Briefly, the fabrication process involved spin-coating a photoresist onto the wafer, exposure to UV radiation through a patterned photomask, and subsequent etching of the silicon wafer using an STS Deep Reactive Ion Etcher device. PDMS membranes were prepared according to the manufacturer’s protocol (Sylgard 184; Dow Corning), involving mixing the base agent with the curing agent, degassing, pouring onto the silicon wafers, spin-coating, degassing, and curing at 100 °C for 1 h. The final membranes were carefully removed and cut to the desired size (2 cm × 2 cm).

### Experimental design and cell culturing on PDMS substrates

After sterilizing the PDMS membranes according to the previous publication, to create a favorable site for the attachment of hMSCs, the micropatterned or flat PDMS substrates were first treated with oxygen plasma at a frequency of 13.56 MHz for 1 min and then coated with collagen type 1 (Sigma-Aldrich, Germany) at a density of 0.1 mg/ml. Initially, 10^4^ cells/cm^2^ was seeded on the micropatterned and flat substrates for 1 d (day 0) in the presence of normal medium. After day 0, the culture medium was replaced with normal medium or the one supplemented with TGF-β1 (10 ng/ml) for 6 d. The culture medium was then changed every 2 d with a fresh medium. Overall, two independent variables were selected: treatment type (normal medium and culture medium containing TGF-β1), and substrate topography (flat and micropatterned substrates). At the experimental time point of 6 d, all samples were prepared for immunoblotting analysis, immunofluorescence staining, reverse transcription quantitative polymerase chain reaction (RT-qPCR), and phase-contrast microscopy.

### Immunofluorescence staining

Cells were fixed in 4% paraformaldehyde and permeabilized with 0.25% Triton X-100 for 10 min, followed by blocking with 5% bovine serum albumin (BSA) and incubating with primary/secondary antibodies. The MHY11 and KLF4 proteins were separately labeled using immunofluorescence staining. To achieve this, the cells were fixed, permeabilized, and blocked as described above. To stain MYH11 protein, the cells were incubated with the primary rabbit polyclonal anti-smooth muscle myosin heavy chain 11 antibody (ab82541) diluted at 1:200 in 2% BSA overnight. The samples were washed with PBS three times and subjected to goat anti-rabbit IgG H&L (FITC) (ab7090) secondary antibody diluted at 1:1000 in 2% BSA for 1 h. After rinsing the cells with PBS three times, they were incubated with 4′,6-diamidino-2-phenylindole (DAPI, Sigma) at a concentration of 1:1000 in PBS. All processes were performed in the dark. For KLF4 immunostaining, the same process was followed, except that the primary antibody was rabbit monoclonal anti-human KLF4 (ab215036). The prepared samples were visualized using Cytation 3™ (BioTek Gen5 software).

### Western blots

At the experimental time points, cell lysates were prepared in modified radioimmune precipitation assay (RIPA) buffer (Santa Cruz Biotechnologies) which contained 1 × tris-buffered saline (TBS), 1% Nonidet P-40, 0.5% sodium deoxycholate, 0.1% SDS, 0.004% sodium azide,10 μl PMSF solution, 10 μl sodium orthovanadate solution, and 10–20 μl protease inhibitor cocktail solution per 1 ml of 1 × RIPA lysis buffer, quantified by Bradford protein assay (Bio-Rad), separated by SDS-PAGE, transferred onto nitrocellulose membranes, and blocked with 5% BSA. The membranes were then incubated with primary antibodies against MYH11 (ab82541, Abcam) and KLF4 (ab215036, Abcam). This was followed by incubation with an HRP-conjugated goat anti-rabbit secondary antibody (goat anti-rabbit IgG H&L (HRP) reabsorbed ab7090). To confirm equal loadings in all lanes, the membranes were also incubated with an antibody against glyceraldehyde 3-phosphate dehydrogenase (GAPDH) (R&D Systems, Bio-Techne) diluted in TBS. Protein bands were visualized using an ECL detection system (Invitrogen, Novex) and quantified using ImageJ.

### Reverse transcription quantitative polymerase chain reaction (RT-qPCR)

Total RNA was isolated using TRIzol reagent (ThermoFisher). The integrity, purity, and concentration of the extracted RNA were evaluated by electrophoresis on a 1% agarose gel and in 260/280 absorption ratios using a NanoDrop spectrophotometer (Aosheng, China). Afterward, reverse transcription (RT) was performed using a reverse transcriptase kit (Thermo Fisher Scientific, USA) to synthesize complementary DNA (cDNA). The StepOne plus™ thermal cycler (ABI) was used to perform RT-qPCR using 2 × real-time PCR master mix high ROX (BioFact, South Korea) in 25-μl reaction volume. The qPCR program included an initial denaturation at 95 °C for 10 min followed by 35 cycles at 95 °C for 25 s, 60 °C for 20 s, and 72 °C for 20 s, followed by the melt curve stage assessment. The primer sequences are presented in Table 1. All experiments were performed in triplicate. GAPDH was used as the internal control.

**Table 1.** Primer sequences used for gene amplification

Gene name	Primer sequence
MHY11	F: TGCTTTCGCTCGTCTTCC R: CGGCAACTCGTGTCCAAC
KLF4	F: CCCAATTACCCATCCTTCCT R: CGTCCCAGTCACAGTGGTAA
CDH11	F: GGCAGCAAGTATCCAATGG R: TTTGGTTACGTGGTAGGCAC
GAPDH	F: CTCTGACTTCAACAGCGAC R: CGTTGTCATACCAGGAAATGAG

### Statistical analysis

All data were analyzed using SPSS 16.0 (SPSS Inc, Chicago, IL) or GraphPad Prism 7.0 (GraphPad Software, LaJolla, CA, USA). Data comparisons were analyzed using a two-tailed unpaired Student’s *t*-test for two groups and a one-way ANOVA for multiple groups, with Tukey’s post hoc test for pair-wise comparisons of means. In real-time PCR, to calculate the efficiency of the amplification reaction and fold-change analysis, LinRegPCR and REST 2009 software were used, respectively. Results are presented as mean ± SEM from at least three independent experiments.

## Results

### Substrate-induced hMSCs elongation and cell behaviors

**Figure 1.**
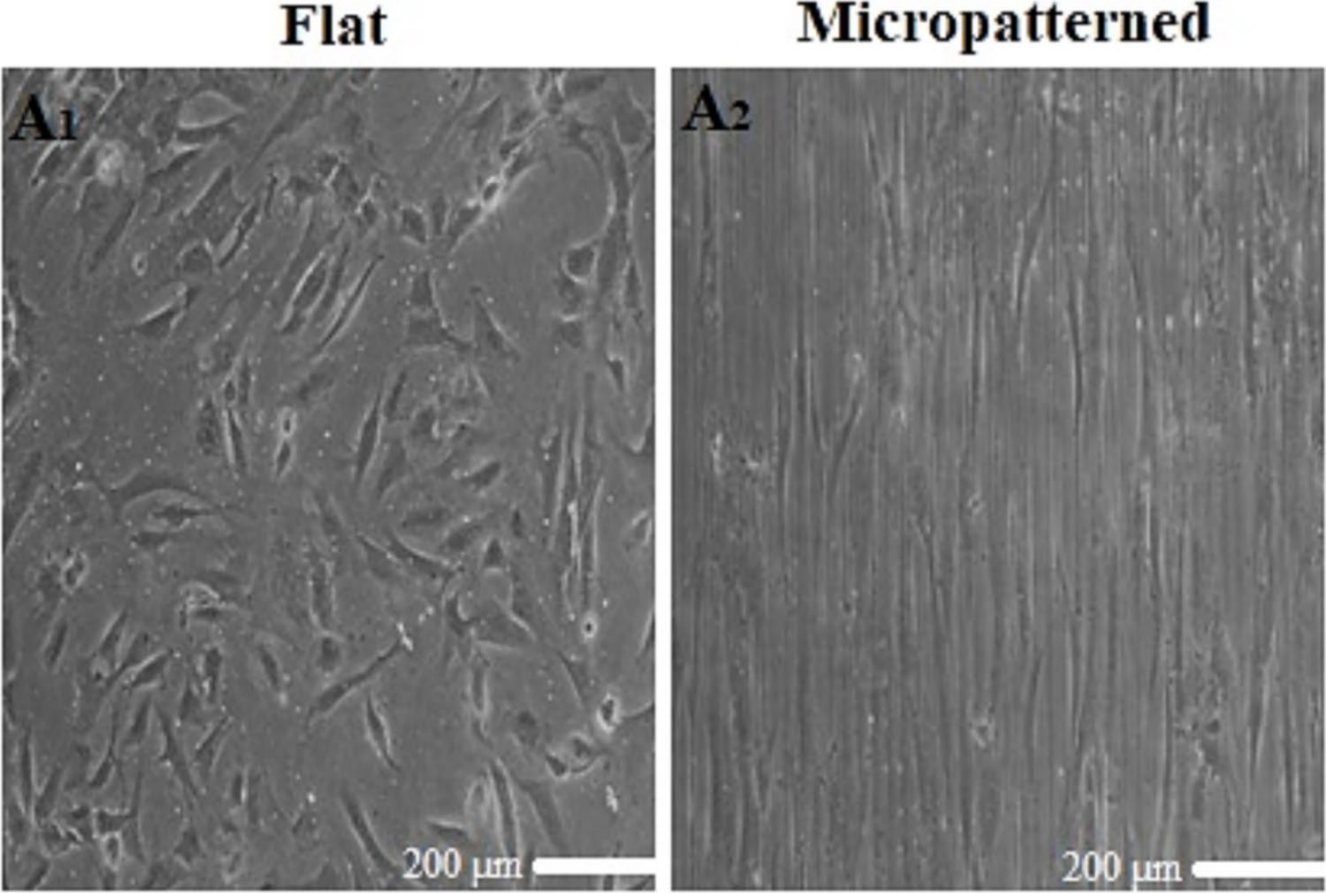
Phase-contrast microscopy images of MSCs. (*A*_*1*_) MSCs cultured on flat PDMS substrates for 6 d display a typical cell morphology. (*A*_*2*_) MSCs on micropatterned PDMS substrates show an elongated cell shape induced by the micropatterns.

The anisotropically patterned cell substrates critical for the differentiation of hMSCs into myogenic cell lineages were used to investigate the molecular mechanism by which the microenvironment promotes the differentiation of hMSCs into SMCs. MSCs seeded on micropatterned substrates without TGF-β1 aligned with the grooves, whereas those on flat substrates displayed random orientations, indicating that substrate topography significantly influences MSC behavior. Microscopic analysis revealed that the hMSCs on anisotropically patterned substrates were highly aligned, highlighting the importance of parallel microgrooved designs in our experimental approach (Fig. 1A_1,2_). The elongated shape induced by the microenvironment significantly promoted SMC differentiation.

### Synergy between substrates and TGF-β1 for SMC differentiation

In this study, we investigated the synergistic effect of cell morphology and TGF-β1 on the differentiation of hMSCs into SMCs by comparing hMSCs cultured on micropatterned and flat substrates with TGF-β1 treatment. Our previous studies have demonstrated that TGF-β1 promotes the differentiation of hMSCs into SMCs on micropatterned substrates after 6 d, as indicated by increased protein expression of SMC markers such as α-smooth muscle actin and h1-calponin (Abolhasani et al. 2020). To confirm the differentiation of hMSCs into SMCs after TGF-β1 treatment and micropatterned substrates, we performed immunoblotting analysis and immunostaining of MYH11 expression.

**Figure 2.**
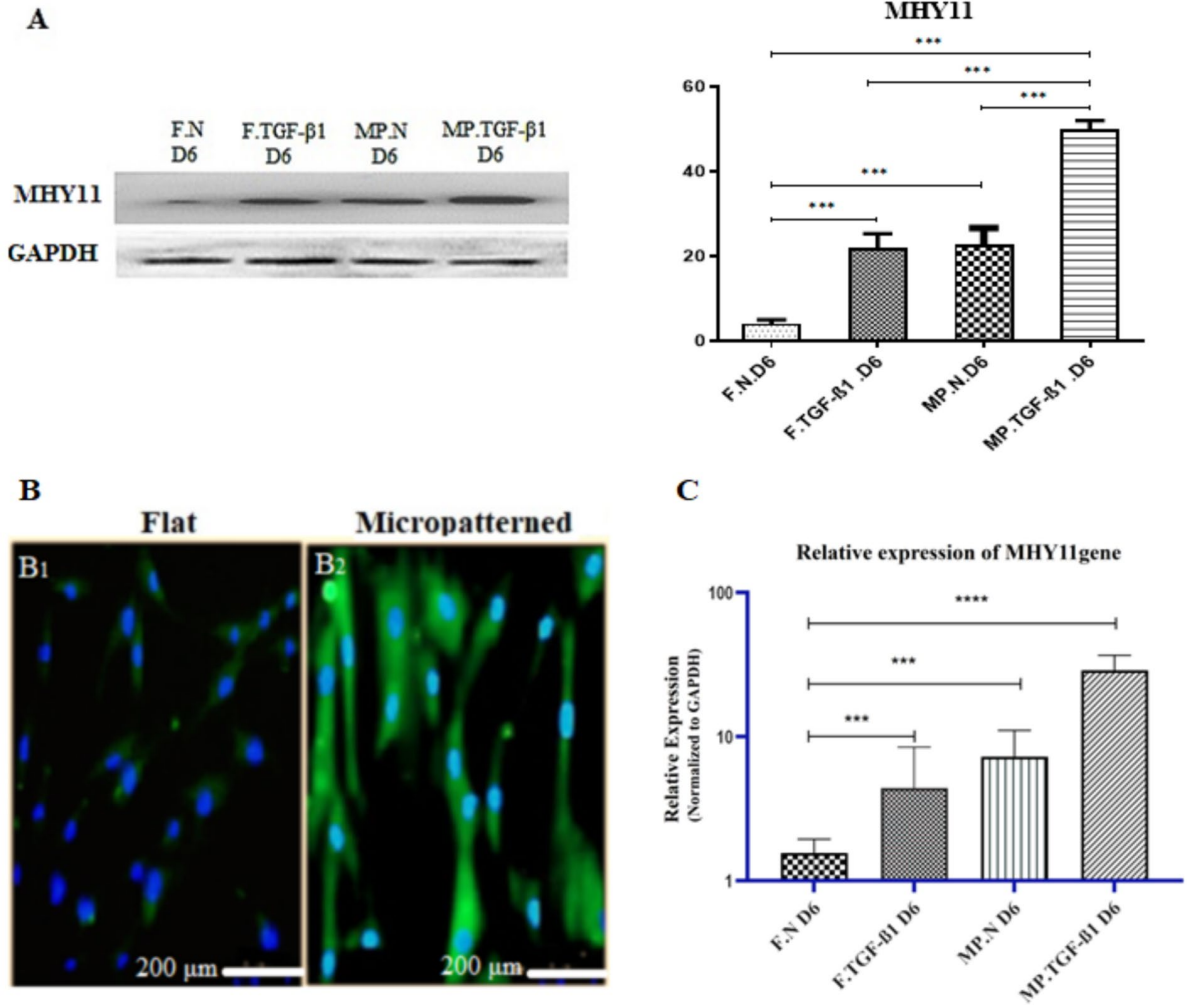
Synergistic effects of micropatterned substrates and TGF-β1 on MYH11 expression. (***A***) The expression of MYH11 protein in MSCs cultured on micropatterned versus flat substrates in normal DMEM medium, with or without TGF-β1, was assessed after 6 d. Glyceraldehyde 3-phosphate dehydrogenase (GAPDH) served as an internal control in all samples. The expression of MYH11 was normalized to that of GAPDH. The data are presented as mean ± SEM of band intensity normalized to MSCs on flat substrates (*n* = 3). (**B**) Immunofluorescence staining of MYH11 and DAPI in MSCs: (*B*_*1*_) MSCs cultured on flat substrates without TGF-β1 show the lowest MYH11 expression. (*B*_*2*_) MSCs on micropatterned substrates with TGF-β1 exhibit the highest MYH11 protein expression as detected by immunoblotting. DAPI staining indicates cell nuclei. (***C***) The expression levels of MYH11 mRNA were evaluated using RT-qPCR. The results are expressed as mean ± SEM, normalized to flat substrates (*n* = 3). Statistical significance was indicated as follows: *p < 0.05, **p < 0.01, ***p < 0.001, ****p < 0.0001. Key abbreviations include *F* (flat substrate), *MP* (micropatterned substrate), *N* (normal medium), and *D6* (day 6). *MYH11* refers to the smooth muscle myosin heavy chain.

The results indicated that hMSCs cultured on micropatterned substrates and treated with TGF-β1 exhibited significantly higher MYH11 protein expression compared with those on flat substrates and micropatterned substrates without TGF-β1, as well as flat substrates with TGF-β1, after 6 d (*p*-value < 0.001 by ANOVA). In addition, hMSCs cultured on micropatterned substrates without TGF-β1 and flat substrates with TGF-β1 exhibited significantly higher MYH11 protein expression than those on smooth substrates without TGF-β1 after 6 d (*p*-value < 0.001 for both). However, no significant differences were observed between the topography of the substrate and the type of treatment (*p*-value = 0.56 according to ANOVA) (Fig. 2A). The upregulation of MYH11 protein expression was also confirmed by immunostaining and RT-qPCR.

Figure 2B1 and B2 show immunostaining of MYH11 expression in the differentiated cells revealing the incorporation of MYH11 protein expression in hMSCs cultured on micropatterned substrates after TGF-β1 stimulation. In contrast, MYH11 protein was not distributed in hMSCs cultured on flat substrates for 6 d without TGF-β1 treatment.

MYH11 mRNA levels were measured using RT-qPCR. After 6 d of culture of hMSCs on micropatterned substrates and TGF-β1 treatment, MYH11 mRNA levels were significantly higher than those in cells cultured on flat substrates without TGF-β1 (*p* < 0.0001) and other groups (*p* < 0.001). Culturing on flat substrates without TGF-β1 suppressed MYH11 upregulation. The expression of MYH11 in hMSCs on micropatterned substrates without TGF-β1 was similar to that in hMSCs on flat substrates with TGF-β1, both significantly elevated compared with those on flat substrates without TGF-β1 (*p* < 0.001) (Fig. 2C).

### Modulation of KLF4 expression by substrates

To explore the mechanism underlying the differential communication of hMSCs to SMCs induced by cell elongation, we examined the protein and mRNA levels of KLF4 in hMSCs cultured on micropatterned or smooth substrates both with and without TGF-β1.

Using immunoblotting, we observed an intriguing result: hMSCs cultured on flat substrates without TGF-β1 expressed significant levels of KLF4, whereas protein levels decreased significantly when cells were exposed to TGF-β1 and micropatterned substrates.

**Figure 3.**
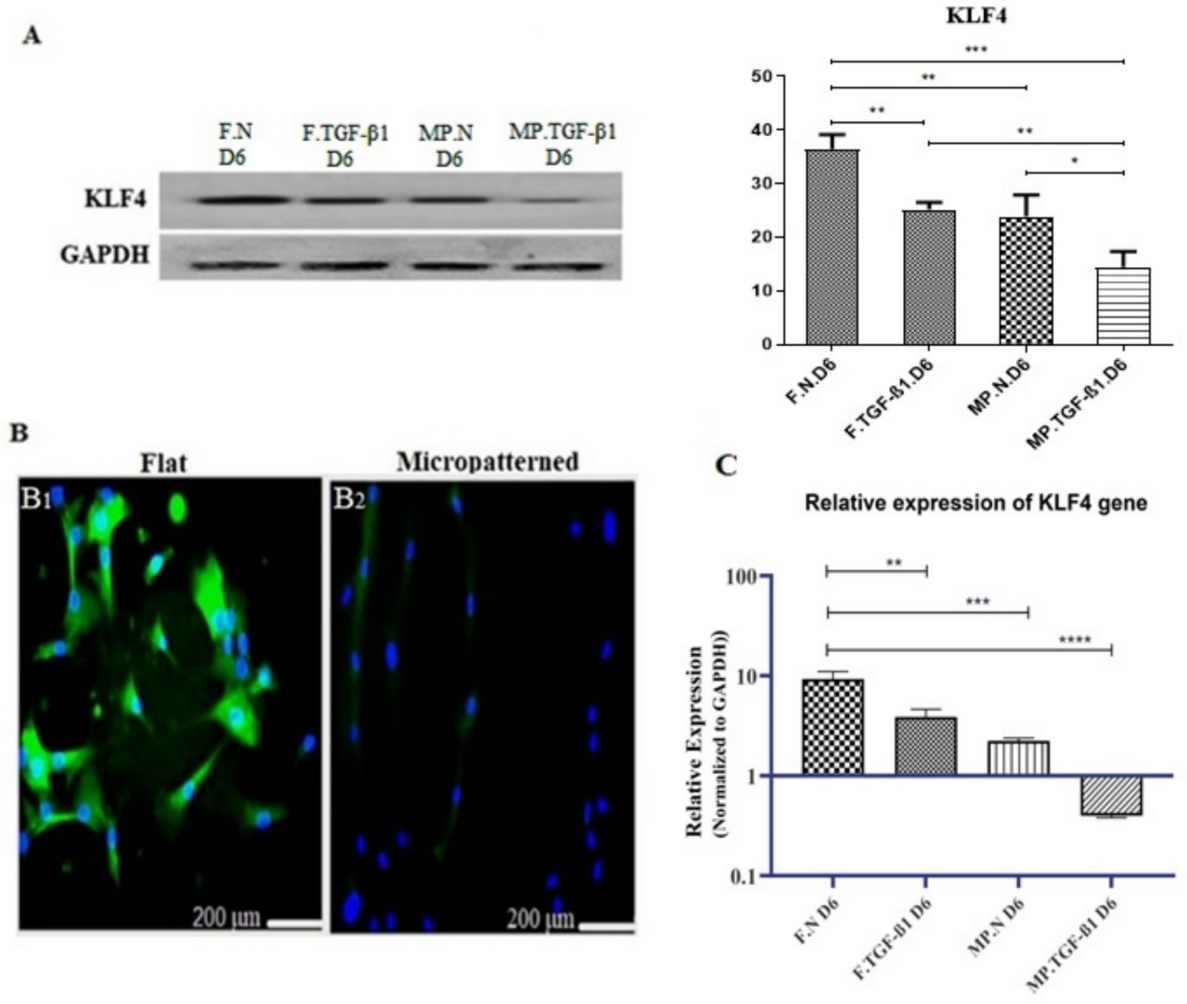
Micropattern and TGF-β1 modulate KLF4 expression in MSCs. (***A***) Immunoblotting analysis of KLF4 in mesenchymal stem cells (MSCs) cultured on micropatterned and flat substrates in normal DMEM, administered with or without TGF-β1 treatment for 6 d. KLF4 protein expression was normalized to GAPDH levels. The data are shown as mean ± SEM of band intensity, with values normalized to MSCs on flat substrates (*n* = 3). (***B***) Immunofluorescence staining of KLF4 and DAPI in MSCs: (*B*_*1*_) MSCs cultured on flat substrates without TGF-β1 exhibit the highest KLF4 protein expression. *(B*_*2*_) MSCs on micropatterned substrates with TGF-β1 show the lowest KLF4 expression as detected by immunoblotting. DAPI staining indicates cell nuclei. (*C*) Relative gene expression analysis of KLF4: KLF4 mRNA levels were quantified via RT-qPCR, normalized against flat substrate controls. Results are presented as mean ± SEM (*n* = 3), with statistical significance indicated as follows: *p < 0.05, **p < 0.01, ***p < 0.001, ****p < 0.0001. Abbreviations used include *F* (flat substrate), *MP* (micropatterned substrate), *N* (normal medium), and *D6* (day 6). *KLF4* refers to Krϋppel-like factor 4.

ANOVA revealed a significant increase in KLF4 protein levels in cells cultured on flat substrates with a normal medium for 6 d, whereas both the treatment type and substrate topography significantly reduced KLF4 protein levels (*p* < 0.01). hMSCs on micropatterned substrates treated with TGF-β1 exhibited the lowest KLF4 expression, whereas those on flat substrates without TGF-β1 showed the highest (*p* < 0.001). Additionally, hMSCs on flat substrates with TGF-β1 and those on micropatterned substrates without TGF-β1 had higher KLF4 expression than hMSCs on micropatterned substrates with TGF-β1 (*p* < 0.01 and *p* < 0.05, respectively) (Fig. 3A).

Immunostaining analysis supported the immunoblotting data and showed that KLF4 was abundant in hMSCs cultured on unpatterned substrates and treated with a normal medium but was significantly reduced when cultured on micropatterned substrates after TGF-β1 stimulation (Fig. 3B1 and B2).

To determine whether micropatterned substrates could downregulate KLF4 expression, we analyzed mRNA levels by RT-qPCR after hMSCs were cultured on micropatterned substrates and treated with TGF-β1 for 6 d. The results showed that the mRNA level of KLF4 was significantly reduced in cells cultured on micropatterned substrates and treated with TGF-β1 for 6 d (*p*-value < 0.0001). Furthermore, there was approximately a similar degree of downregulation of KLF4 mRNA in cells cultured on micropatterned substrates without TGF-β1 and those cultured on unpatterned substrates in the presence of TGF-β1 compared to cells cultured on unpatterned substrates and treated with a normal medium for 6 d (*p*-value < 0.01 and 0.001 respectively). hMSCs on micropatterned substrates with TGF-β1 exhibited significant downregulation of KLF4 mRNA compared with hMSCs on flat substrates with TGF-β1 (*p* < 0.01) and those on micropatterned substrates without TGF-β1 (*p* < 0.05) (Fig. 3C). Taken together, these results suggest that substrate topography and TGF-β1 treatment can downregulate KLF4 in hMSCs and inhibit KLF4 expression to induce contractile gene expression.

### Elongation and development of focal adhesion sites

**Figure 4.**
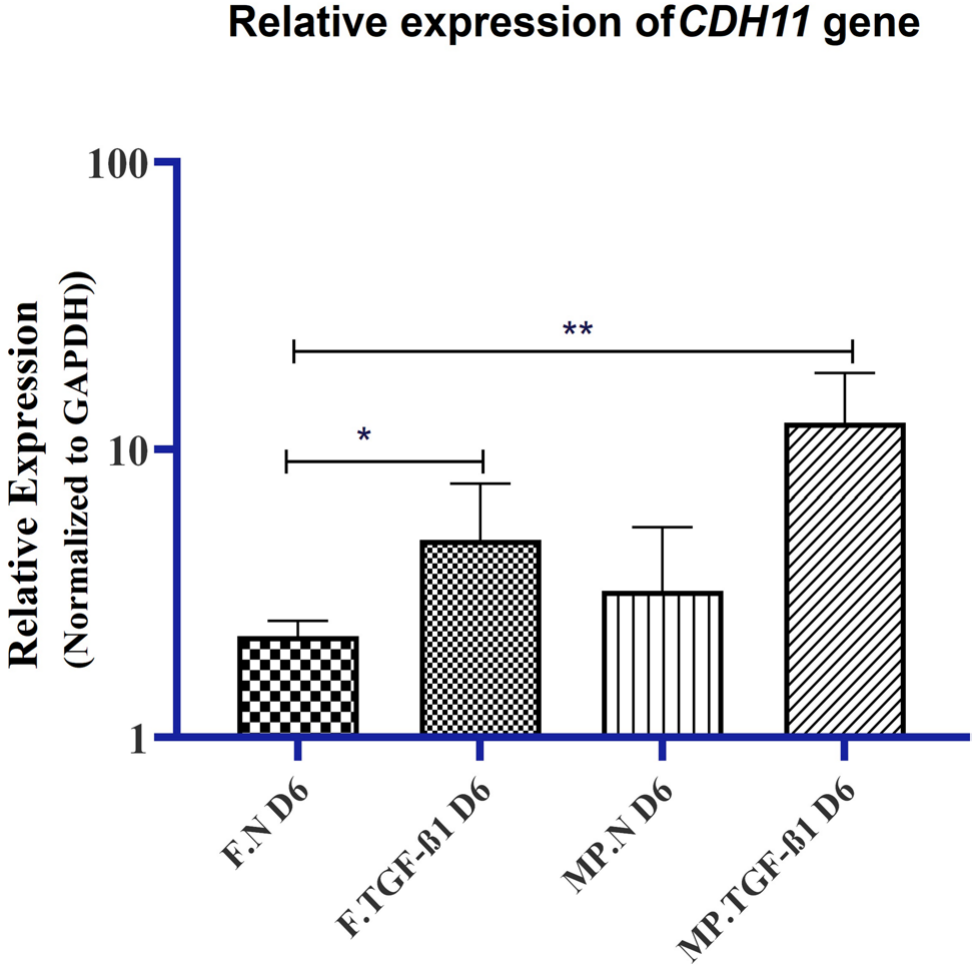
Elongation and TGF-β1 regulate cadherin-11 gene. Relative gene level expression analyses of cadherin-11. The expression of cadherin-11 mRNA was examined by RT-qPCR. The graphic data is mean ± SEM of the value normalized to flat substrates (*n* = 3). **p < 0.01. The terms *F*, *MP*, *N*, and *D6* represent a flat substrate, a micropatterned substrate, a normal medium, and day 6, respectively.

This process is mediated by the participants’ interaction with the ECM. The way in which cells expand plays an important role in controlling this development. Cadherin-11, a cell adhesion molecule expressed by hMSCs, has been identified as a key factor for development into the myocyte’s lineage. The mRNA levels of cadherin-11 were measured in the hMSCs cultured on micropatterned substrates and treated with TGF-β1 for 6 d. As shown in Fig. 4, the mRNA levels of cadherin-11 were significantly increased in hMSCs cultured on micropatterned substrates and treated with TGF-β1 compared to those cultured on smooth substrates and not treated with TGF-β1 (*p*-value < 0.01). In addition, hMSCs treated with TGF-β1 and cultured on flat substrates showed significant upregulation of cadherin-11 mRNA levels compared with those cultured on micropatterned and flat substrates and not treated with TGF-β1 (*p*-value < 0.05). HMSCs seeded on micropatterned substrates exhibited an insignificant increase in cadherin-11 mRNA levels compared with those seeded on flat substrates without TGF-β1 (*p*-value < 0.73). These results indicate that the elongation configuration and TGF-β1 treatment, which control the development of focal adhesion (FA) sites, play an essential role in the differentiation of MSCs into SMCs.

Overall, our results suggest that the combination of micropatterned matrices and TGF-β1 treatment effectively promotes the differentiation of hMSCs into VSMCs. This is accompanied by changes in the expression levels of Krϋppel-like factor 4 and the upregulation of cadherin-11 and MYH11 expression.

## Discussion

The combined effect of micropatterned substrates and TGF-β1 on the differentiation of hMSCs into VSMCs has been extensively studied (Ding et al. 2020; Tay et al. 2015; Yeh et al. 2019). However, in our study, substrates with groove widths were used as an excellent platform for the conversion of elongated hMSCs into contractile SMCs, although the mechanisms underlying SMC differentiation induced by MSC elongation have not yet been clearly elucidated. Our results show that the interplay between these two factors significantly enhances the efficiency and maturation of hMSCs into VSMCs. This differentiation is accompanied by changes in the expression of key genes involved in this process, including Krϋppel-like factor 4 (KLF4), cadherin-11 (CDH11), and MYH11.

The shape of a cell often changes before it develops into a particular type, suggesting that cell shape can influence stem cell differentiation (Engler et al. 2006; McBeath et al. 2004). When cell substrates are patterned certainly, MSCs show visible elongation and adopt a fibroblast-like shape (Kasalkovova et al. 2024). The elongated nuclear shape of MSCs leads to increased histone acetylation, which regulates cell function and promotes smooth SMC differentiation (Li et al. 2011). Microscopic examination revealed that the hMSCs were highly aligned on the microgroove substrates (Fig. 1A1 and A2), suggesting that designing the culture substrates with parallel microgrooves is crucial for the experimental approach. This alignment is crucial for the differentiation of cells into smooth muscle cells because it enables the proper organization of α-smooth muscle actin (α-SMA) on the existing stress fibers (SFs) and the generation of an intermediate contractile force with an appropriate cytoskeletal architecture (Yeh et al. 2019). In addition, our previous study showed that elongated cell morphology leads to cytoskeletal reorganization and generates optimal cellular tension to induce SMC binding to the cell line (Abolhasani et al. 2020). Furthermore, previous studies have shown that the Rho/ROCK signaling pathway plays an important role in cytoskeletal remodeling and modulation of intracellular tension in stem cells, which are essential for determining their developmental pathway (McBeath et al. 2004; Ribeiro et al. 2024). It has been reported that activation of the Rho/ROCK pathway increases cytoskeletal tension and cytoskeletal reorganization promotes the differentiation of MSCs into myogenic cells (Kilian et al. 2010). Moreover, the elongated shapes of cells and nuclei induced by Rho/ROCK signaling contribute significantly to the differentiation of MSCs into SMCs (Eyckmans et al. 2012; Yeh et al. 2019). The rationale behind this is that an environment that resembles the natural niche of SMCs can promote the differentiation of MSCs into SMCs with enhanced functionality that closely resembles the characteristics of native SMCs (Yogi et al. 2021). Finally, this VSMC phenotype was achieved in as little as 1 d. It remained stable for up to 14 d (data not shown), regardless of whether the hMSCs were maintained in differentiation or switched to normal media. This stable phenotype is significant because previous reports indicated that the acquired phenotype may be transient in certain differentiation protocols (Ning et al. 2009). This suggests that the cells retain the contractile properties of SMCs and thus have great potential for use in tissue engineering applications.

The synergistic effects of cell morphology and TGF-β1 on the differentiation of hMSCs into SMCs were investigated by comparing hMSCs cultured on micropatterned and flat substrates with TGF-β1 treatment. The results showed that MSCs cultured on micropatterned substrates and treated with TGF-β1 had significantly higher MYH11 protein expression than those cultured on flat substrates without TGF-β1. As previously shown, TGF-β1 promotes the differentiation of hMSCs into SMCs on micropatterned substrates. The increased protein expression of SMC markers, such as α-smooth muscle actin and h1-calponin, indicates this promotion (Abolhasani et al. 2020; Nakamoto et al. 2014). Furthermore, hMSCs cultured on micropatterned substrates and treated with TGF-β1 showed significantly higher MYH11 protein expression compared to those on flat substrates without TGF-β1. Interestingly, the combination of micropatterned substrates and TGF-β1 treatment had a greater impact on the expression of MYH11 than individual stimuli. The upregulation of MYH11 protein expression was confirmed by immunostaining and RT-qPCR analysis (Fig. 2B_1,2_ and *C*). The potential for improving vascular grafts with improved mechanical properties and physiological relevance is incredibly promising (Yeh et al. 2019). Therefore, our research provides strong evidence that the synergistic application of micropatterned substrates and TGF-β1 treatment substantially enhances the expression of MYH11, a mature marker of SMC-specific contractile protein. Moreover, the role of synthetic markers is crucial for a comprehensive understanding of SMC differentiation. Synthetic SMCs are characterized by increased proliferation and enhanced ECM protein production. Key markers include cadherin-11, which is influenced by micropatterned substrates and TGF-β1 treatment, indicating its role in SMC differentiation (Mansouri et al. 2019); collagen type I, which is often upregulated in synthetic SMCs and important for ECM remodeling (Gubbiotti et al. 2017); and fibrillin, which is associated with elastic fiber formation during vascular remodeling (Xu et al. 2021). To gain deeper insights into the differentiation process, it is necessary to incorporate these markers into the analysis.

The results of the present study show that KLF4 protein expression is significantly higher in hMSCs cultured on flat, TGF-β1-free substrates than in those seeded on micropatterned substrates with TGF-β1 treatment. Analysis of variance (ANOVA) confirmed the statistical significance of this difference (*p*-values 0.001), indicating a robust effect of substrate topography and TGF-β1 on KLF4 expression. This suggests that the role of KLF4 in committing to an SMC phenotype may be contradictory in two distinct settings, potentially implicating its ability to promote fibroblast-like behavior on micropatterned substrates (Aji et al. 2017). Interestingly, immunostaining further supports these findings, showing that KLF4 levels decrease with TGF-β1 treatment and microgrooved substrates, which correlates with increased MYH11 expression. This finding supports the idea that lower KLF4 levels facilitate the activation of smooth muscle-specific genes (Aji et al. 2017; Yeh et al. 2019). However, it is important to consider the possibility of a bidirectional relationship, in which differentiation into VSMCs may also cause intrinsic changes in KLF4 expression, independent of external factors. Future studies should explore this reciprocal regulation in greater detail to elucidate its mechanisms. The study aimed to understand the impact of micropatterned substrates on cells. RT-qPCR measurements were performed, revealing a noteworthy decrease in KLF4 mRNA levels in cells exposed to TGF-β1 for 6 d, regardless of whether they were on micropatterned or unpatterned substrates (Fig. 3C). This decrease in KLF4 mRNA was consistent under both conditions, indicating that the effect was not solely due to changes in the substrate but also due to the combination of TGF-β1. Previous studies have demonstrated that TGF-β1 and BMP4 can downregulate KLF4, leading to smooth muscle differentiation (Aji et al. 2017). Additionally, it has been demonstrated that PDGF-BB induces the expression of KLF4 by activating its receptor and the subsequent transcription factor Sp1. Interestingly, Sp1 also increases the expression of *ACTA2* through the signaling of TGF-β1 (Sivaraman et al. 2021). TGF-β1 triggers the production of VSMC contractile proteins by activating specific members of the SMAD transcription factor family, which promotes the VSMC contractile phenotype (Sivaraman et al. 2021; Yap et al. 2021).

Furthermore, KLF4 influences both stem cell pluripotency and SMC phenotypic switching under microgrooved topography induction (Shankman et al. 2016; Zhang et al. 2010). Our experiments support the idea that microgroove substrates induce lower KLF4 expression in hMSCs than flat substrates. Biomaterial cues like topography act as potent regulators of epigenetic states and cell functions (Lv et al. 2018). Recent studies have suggested that the inhibition of KLF4 by miR-145 enhances the differentiation of MSCs into SMCs in response to cell elongation, indicating that KLF4 plays multiple roles in regulating the SMC phenotype (Yeh et al. 2019).

In particular, micro-grooved substrates limited MSC spreading, increased cytoskeletal tension, and caused MSC elongation in a direction- and width-dependent manner (Yap et al. 2021). The elongated cell shape led to SMC differentiation through the miR-145/KLF4 pathway. miR-145 overexpression downregulated KLF4 expression, and this reduction of KLF4 was abolished by anti-miR-145 (Yeh et al. 2019). These findings suggest that topographical cues likely induce epigenetic changes that regulate the expression of KLF4 and downstream contractile genes (Yeh et al. 2019). This provides a potential mechanism by which biomaterial topography can direct the VSMC phenotype and function (Lu et al. 2024; Xin et al. 2009). This indicates that controlling KLF4 is a key mechanism for efficiently differentiating hMSCs into functional VSMCs. Downregulation of KLF4 expression is crucial for contractile gene expression and differentiation of hMSCs into VSMCs. Micropatterned substrates and TGF-β1 treatment effectively regulate KLF4 expression to drive this differentiation process. Further research into specific signaling pathways and the role of miR-145 could provide additional insights into KLF4 regulation during the MSC-to-SMC transition.

Micropatterned substrates and TGF-β1 significantly impact the extracellular matrix (ECM) and cellular behavior, with integrins and FAs being crucial in these processes. A recent study showed that cells on micropatterned substrates enhance the synthesis of sulfated glycosaminoglycans (sGAGs), resulting in increased ECM deposition and substrate stiffness over time (Bhattacharjee et al. 2020). Micropatterning also helps organize ECM components and create a more native environment (Cady et al. 2020). Moreover, TGF-β1 is a key cytokine that influences differentiation and apoptosis, with its effects varying based on substrate stiffness; it can induce apoptosis on compliant substrates and promote epithelial-mesenchymal transition (EMT) on rigid substrates, altering cell morphology and increasing mesenchymal marker expression (Leight et al. 2012). Correspondingly, integrins mediate cell adhesion to the ECM and form FAs, which act as mechanosensors that respond to substrate properties, influencing signaling pathways that regulate cell behavior in response to TGF-β1 (Verma et al. 2023). These adhesion molecules are essential for the differentiation of hMSCs into VSMCs and affect proliferation, migration, and differentiation (O'Connor 2022). Cadherins also contribute to cell adhesion through cell–cell interactions, and their relationship with integrins is vital for maintaining tissue integrity and responding to mechanical and biochemical signals, thereby impacting ECM remodeling and cellular differentiation in tissue engineering (Frismantiene et al. 2018). In the present study, the development of FA sites in hMSCs during their differentiation into SMCs was investigated. In the differentiation of hMSCs into VSMCs, the process of elongation and the formation of FA sites are important factors influenced by the interaction with the ECM (Alimperti et al. 2014; Niessen et al. 2011). The extent to which the cells elongate significantly influences the direction of this developmental pathway, cadherin-11, a cell adhesion molecule expressed by MSCs, which is crucial in directing their orientation toward the myocyte lineage (Park et al. 2022). The study focused on the changes in cadherin-11 gene expression in hMSCs in response to specific environmental cues. RT-qPCR was used to analyze cadherin-11 mRNA levels in hMSCs cultured on micropatterned substrates and treated with TGF-β1 for 6 d. The results presented in Fig. 4 showed a significant increase in cadherin-11 mRNA levels in hMSCs cultured on micropatterned substrates and treated with TGF-β1. Additionally, hMSCs treated with TGF-β1 and cultured on flat substrates showed a noticeable upregulation in cadherin-11 mRNA levels. Interestingly, there was a slight increase in cadherin-11 mRNA levels in hMSCs seeded on micropatterned substrates compared with those seeded on flat substrates without TGF-β1 treatment (*p*-value < 0.73). Consistent with our findings, a recent study demonstrated that cadherin-11 expression is upregulated by exogenous TGF-β1 supplementation in myofibroblasts (Xu et al. 2018). The results indicate that biochemical cues play a more important role than biophysical cues in the formation of focal adhesion sites. The results suggest that cells may not be able to develop the necessary FAs, similar to those in the body, when cultured on in vitro substrates. These results emphasize the important role of elongation configuration and TGF-β1 treatment in controlling the development of focal adhesion sites, which ultimately influence the differentiation of hMSCs into the SMC lineage (Alimperti et al. 2014; Park et al. 2022). The observed increase in cadherin-11 mRNA levels under specific culture conditions accentuates the importance of these factors in directing MSC differentiation toward an SMC fate. These findings suggest a promising area for further research into the molecular mechanisms underlying cell adhesion dynamics and lineage commitment in the context of VSMC differentiation from hMSCs.

## Conclusion

It was found in this research that the use of micropatterned substrates along with the application of TGF-β1 to hMSCs is a powerful approach for enhancing the transformation of hMSCs into VSMCs. There was a significant enhancement in the efficacy and maturity of the differentiation of hMSCs to VSMCs with the combined effects of micropatterned substrates and TGF-β1. The change in the expression of important regulatory genes involved in the differentiation process, such as KLF4, CDH11, and MYH11, indicates that the silenced KLF4 promotes the expression of contractile genes that ultimately differentiate hMSCs into VSMCs. The regulation of KLF4 expression by micropatterned substrates and TGF-β1 represented a crucial step in modulating this differentiation step. The cadherin-11 mRNA level changed under certain culture conditions, particularly when MSCs were cultured on micropatterned substrates and treated with TGF-β1, even when compared with control substrates, highlighting the importance of these two factors in driving hMSCs differentiation toward an SMC fate. The combined use of patterned surfaces and TGF-β1 greatly improved the induction of MYH11, a marker of mature SMC-specific contractile protein, by modifying KLF4 expression and increasing cadherin-11 expression. This research underscores the importance of both physical and biochemical cues in stem cell differentiation, paving the way for the exploration of cellular engineering.

## Supplementary Information

Below is the link to the electronic supplementary material.

Supplementary file1 (DOCX 125 KB)

## Data Availability

The raw data supporting the conclusions of this article will be made available by the authors upon reasonable request. This ensures transparency and allows for further validation and exploration of the findings presented in the study.
