# Supplementary material for: Synergistic effects of micropatterned substrates and transforming growth factor-β1 on differentiation of human mesenchymal stem cells into vascular smooth muscle cells through modulation of Krϋppel-like factor 4
Source: In Vitro Cell Dev Biol Anim. 2025 May 23;61(6):644–55. doi: 10.1007/s11626-025-01033-2 (PMC12307518; doi:10.1007/s11626-025-01033-2)
Supplement: Supplementary file 1 — Supplementary file1 (DOCX 125 KB) [file 11626_2025_1033_MOESM1_ESM.docx]

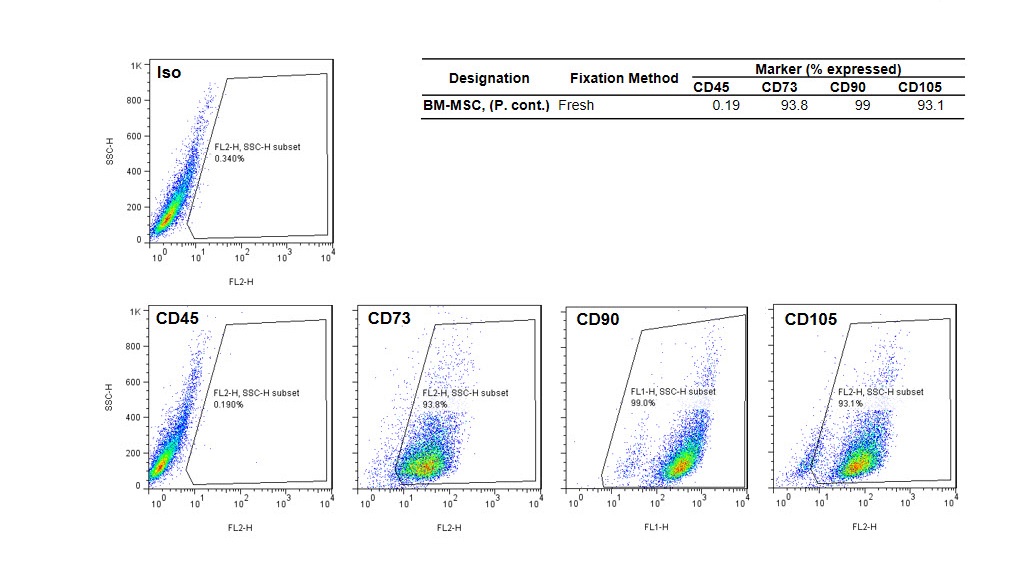


Flow cytometry results confirm the presence of mesenchymal stem cells (MSCs) characterized by the surface markers CD105+, CD90+, CD73+, and CD45-. The surface antigen details were verified through flow cytometry analysis, as indicated in the institution's data sheet.
